# Risk compensation and face mask mandates during the COVID-19 pandemic

**DOI:** 10.1038/s41598-021-82574-w

**Published:** 2021-02-04

**Authors:** Youpei Yan, Jude Bayham, Aaron Richter, Eli P. Fenichel

**Affiliations:** 1 Yale University, 195 Prospect St., New Haven, CT 06511 USA; 2 Colorado State University, B303 Clark Bldg, Fort Collins, CO USA

**Keywords:** Infectious diseases, Environmental economics, Health policy, Health care economics

## Abstract

Face masks are an important component in controlling COVID-19, and policy orders to wear masks are common. However, behavioral responses are seldom additive, and exchanging one protective behavior for another could undermine the COVID-19 policy response. We use SafeGraph smart device location data and variation in the date that US states and counties issued face mask mandates as a set of natural experiments to investigate risk compensation behavior. We compare time at home and the number of visits to public locations before and after face mask orders conditional on multiple statistical controls. We find that face mask orders lead to risk compensation behavior. Americans subject to the mask orders spend 11–24 fewer minutes at home on average and increase visits to some commercial locations—most notably restaurants, which are a high-risk location. It is unclear if this would lead to a net increase or decrease in transmission. However, it is clear that mask orders would be an important part of an economic recovery if people otherwise overestimate the risk of visiting public places.

Most states in the United States require some use of face masks in public, in businesses, or both to combat the COVID-19 pandemic. Similar regulations exist around the world. Prior to the COVID-19 pandemic there was scant evidence that mask-wearing by the general public reduced transmission of coronaviruses during a pandemic. The airborne nature of SARS-CoV-2 suggests masks could reduce spread^1^, and a growing body of evidence supports that high rates of mask use in the general population reduces overall virus transmission^2–4^, despite the lack of any randomized control studies to definitively demonstrate that masks reduce transmission of SARS-CoV-2^4^. Yet, mask quality matters^2,5^, which implies the benefits of face masks depend on correct usage. This latter point highlights that masks are a behavioral intervention, so how they are worn and how they affect other behaviors is as important as the physical attributes of the mask.

The arguments in favor of masks claim that they do no harm, are low cost, and they may provide benefits^6^. However, the cost of face mask orders is more than the price of a mask. Most recommendations are for cloth masks and non-medical masks because of concerns that the opportunity cost of masks might be high, given the scarcity of masks for medical personnel^7,8^. Evidence suggests cloth masks are less effective than N95 respirators^2,5^. A more insidious cost exists if individuals substitute mask wearing for other protective behaviors, risk compensation. If people spend more time in public spaces because they wear masks and believe they are protected, then recommendations to wear masks in public may create a perverse incentive. Furthermore, messaging around wearing masks could create a false impression of safety. A corollary is that mask orders may provide confidence to help people re-engage as the pandemic is controlled if people otherwise overestimate the risk of infection.

Substituting face mask wearing for staying home may increase system-wide risk. If the purpose of wearing face masks were only to protect the wearer from COVID-19 and infection risks were well-estimated, then going out with a mask could be privately optimal so long as masks provide protection equivalent to the forgone minutes at home. It is unlikely, however, that individuals fully account for the costs of their own illness in terms of hospital congestion and other externalized costs. Importantly, the benefit of mask-wearing or staying home is that both prevent the spread of COVID-19 by infectious and potentially asymptomatic individuals. Therefore, wearing a mask could create an un-internalized cost if masks do not sufficiently prevent asymptomatic individuals from spreading the pathogen. Just as wearing a seat belt can encourage less safe driving while conferring no protection to others from such actions, if masks do not sufficiently prevent infectious individuals from spreading the pathogen, then they induce an externality^9^. This is a concern because of the oft-cited externality associated with pathogen spread and protective behavior—people typically fail to account for how their behavior prevents spreading the pathogen to others^10^. The theory of social distancing suggests that if people can mitigate disease risks by lower private cost means, then they will distance less^11^.

The concern of inducing risky behaviors by providing some protection is well established in the broad literature on behavior, risk, and externalities^12^. The public health literature uses the term “risk compensation” to describe the case when someone increases certain risky behavior when using protective equipment^13^. A close analog is condom use and HIV transmission. In the case of risk compensation, people using condoms engage in more sexual activity and increase the risk to susceptible individuals in the population^9,14^. Public health researchers and economists have long been concerned about the behavioral impacts of introducing partially effective prophylaxis or vaccine for viruses such as HIV^15,16^.

The study’s objective is to contribute to the public health literature addressing COVID-19 by using the variation in face mask mandates along with mobile device data to measure the change in the amount of time Americans stayed at home, and the number of visits Americans made to public places, following face mask mandates. This study investigates the substitution effect between two COVID-19 behavioral interventions, staying home (or visiting certain types of businesses) and public face mask mandates. We find evidence of risk compensation behavior as people spend less time at home and make more trips to public places. Though the ultimate impact for face mask orders on transmission depends on the unknown relative effectiveness in breaking transmission of face masks and staying home. These policies are hard to separate empirically because they are often implemented together.

## Materials and methods

**Figure 1:**
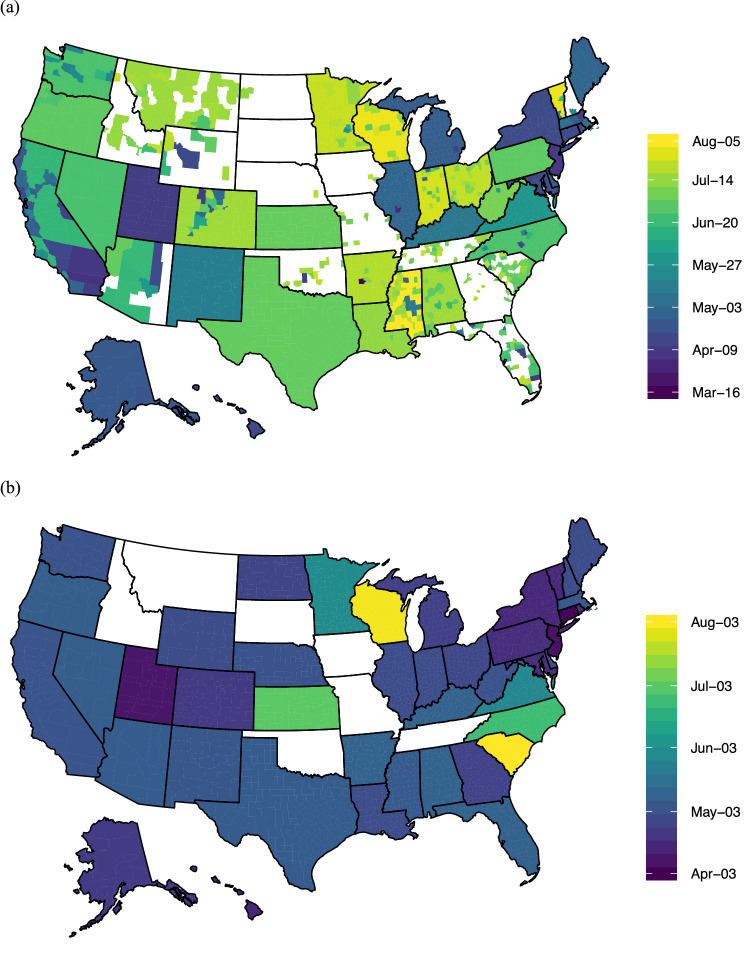
Color scaled dates for face mask mandates (**a**) for public and (**b**) for business. (*Source* for (**a**) https://www.austinlwright.com/covid-research; for (**a**) and (**b**) https://github.com/USCOVIDpolicy/COVID-19-US-State-Policy-Database).

As of August 22, 2020, 42 US states mandated face mask use by employees in public-facing businesses, and 48 states ordered all individuals in public spaces to wear face masks^17,18^) (see Fig. 1). We examine the two policies separately when possible. We use SafeGraph home dwell time and public location visitation data to evaluate the effect that face mask orders had on representative behaviors that could expose individuals to COVID-19 transmission. SafeGraph is a data company that aggregates anonymized location data from numerous smart device applications in order to provide insights about physical places, via the Placekey Community. To enhance privacy, SafeGraph excludes census block group information if fewer than five devices visited an establishment in a month from a given census block group. (https://docs.safegraph.com/docs/social-distancing-metrics). SafeGraph reports the median home dwell time by Census Block Group, and we produce a device weighted average for each county. We also calculate device-weighted averages of trip visits to points of interest by four-digit NAICS (North American Industry Classification System) code.

### Time at home

We examine behavior aligned to 2 weeks before and after each of the counties or states implemented face mask mandates. For a county ${i}$ on day $t$, we regress time spent at home measured as the device-weighted county mean of the median Census Block Group home dwell time in minutes, $Y_{it}$, on the first mask mandate for the public, $M1_{it}$, and on the first mask mandate for use in businesses, $M2_{it}$. We condition the regression on reported cases and newly increased cases in one’s own county, state, and nationwide, $C_{it}$ ^19^, on other county-level controls, $X_{it}$ (including weather, the device count by Safegraph for each county, and holiday dummies), a county-specific fixed effect, $a_{i}$, and a weekday specific fixed effect, $w_{t}$. We consider the possibility that individuals have become exhausted with stay-at-home orders, distancing fatigue^20^, by including the log of days since a stay at home order was implemented, $S_{it}$. The two face mask mandates are generally after the stay-at-home policy is issued but can be before or after the end of the stay-at-home policy (see Figures S1-3). We include an additional group of policy variables to examine the possible effects of various business reopening in some states. The vector, $R_{it}$, includes policy dummies of businesses, daycares, bars, restaurants, movie theaters, hair salons, religious opening, non-essential retailers, and gyms being allowed to reopen, as well as a dummy for the end of stay-at-home policy.

Weather variables are constructed by aggregating 4 km gridded estimates of maximum and minimum daily temperature, maximum and minimum relative humidity, precipitation amount, surface solar radiation, and wind speed^21^. We cluster standard errors at the state level to account for state-level serial correlation and heteroscedasticity caused by the phase-in of orders and because for most states there is very little variation at the county level. The model is specified as

1 $$Y_{it} = \alpha + \gamma_{1} M1_{it} + \gamma_{2} M2_{it} + \delta R_{it} + \beta_{0} ln \left( {C_{it} + 1} \right) + \theta ln \left( {S_{it} + 1} \right) + \rho X_{it} + a_{i} + w_{t} + \epsilon_{it}$$

If people attempt to manage infection risk by substituting the use of masks for time at home, we hypothesize that people spend less time at home once they receive a directive to wear masks, $\gamma_{1} < 0$ and $\gamma_{2} < 0$. Thus, $\gamma_{1}$ and $\gamma_{2}$ are the parameters of interest, and the remaining terms help eliminate confounding by other variables and if these other terms were not included the regression could violate the standard regression assumption that the expectation of $\epsilon$ it is zero.

We then examine the pre-trend of the policy given the specification. We adopt the dynamic event study model by using a list of policy indicator variables for 2 weeks before and after the face mask mandates in public, $M1_{Dit} (M1_{Dit} = 1$ if $t - D = M1_{it}$). The equation for the face mask use mandates in businesses is similar and constructed by replacing $M1_{Dit}$ with $M2_{Dit}$:

2 $$\begin{aligned} { }Y_{it} { } & = \alpha + \gamma_{{1{\text{D}}}} \mathop \sum \limits_{D = - 13}^{ - 2} M1_{Dit} + \gamma_{{1{\text{D}}}} \mathop \sum \limits_{D = 0}^{14} M1_{Dit} + \gamma_{2} M2_{it} + \delta R_{it} + \beta_{0} ln{ }\left( {C_{it} + 1} \right){ } \\ & \quad + \,{\uptheta }ln{ }\left( {S_{it} + 1} \right){ } + \rho X_{it} + a_{i} + w_{t} + \epsilon_{it} \\ \end{aligned}$$

Equation (2) is similar to Eq. (1), but rather than providing a single slope estimate associated with the mandate, the structure of Eq. (2) allows for a non-parametric day specific impact of the mask mandate, with the other terms playing a similar role as in Eq. (1).

We remove the first and the last treatment indicators before the policy to avoid under-specification^22^. We report results for regressions on the $Y_{it}$ and on $ln(Y_{it} )$ for the above equations. In the Supplemental material, we examine pairs of states with and without orders and analyze these pairs with a difference-in-difference design. These analyses support the overall conclusion, but also suggest the risk compensation result is complex because not all pairs support the risk compensation hypothesis.

### Points of interest visitation

If people decrease their time at home, they must go somewhere. It is important to know if they allocate time to relatively high risk or low-risk locations. Benzell et al.^23^ argue that gyms and grocery stores are relatively high risk and hardware stores, sporting goods stores, and general merchandise stores are moderate-risk locations. Conversely, parks may be relatively low-risk locations. To explore the impact of the mandate of face mask use to site visits, we use point-of-interest (POI) data from January to August 18, 2020 that are within 2 weeks before and after the mask mandates, from SafeGraph to examine the change in visits after the face mask mandate.

We aggregate each day $t$’s visits to a site in industry $I$ located in county $i$, $V_{Iit}$. Each industry type is analyzed independently. $I$ is defined by the first four digits of a location’s NAICS code. We regress the county-aggregated visits, $V_{Iit}$, on the mask mandates, $M1_{it}$ and $M2_{it}$. Similar to Eq. (1), we condition the regression on county-level controls, $X_{it}$, on re-opening dummies $R_{it}$, on reported cases and newly increased cases in the POI’s own county, state, and nationwide, $C_{it}$, on time since the stay-at-home order, $S_{it}$, a county fixed effect $a_{i}$, and a weekday fixed effect $w_{t}$.

3 $$V_{Iit} { } = \alpha + \gamma_{1} M1_{it} + \gamma_{2} M2_{it} + {\updelta }R_{it} + \beta_{0} \ln \left( {C_{it} + 1} \right) + {\uptheta }ln{ }\left( {S_{it} + 1} \right){ } + \rho X_{it} + a_{i} + w_{t} + \epsilon_{it}$$

We focus on the sites in the selected industries in wholesale trade (NAICS sectors #41 and 42), retail trade (NAICS sectors #44 and 45), entertainment and recreation (NAICS sector #71), and accommodation and food services (NAICS sector #72). Consistent with the analysis of time at home, we examine the impact of $M1_{it}$ and $M2_{it}$ before and after 14 days of the mask mandate. Like in Eq. (1) the focus is on $\gamma_{1}$ and $_{{}} \gamma_{2}$, with the other terms playing a similar role to the extra terms in Eq. (1).

## Results

We find evidence that masks are associated with risk compensation behavior and that Americans spend less time at home when living with a face mask mandate. Furthermore, we find weak evidence that Americans spend more time in moderate to high-risk locations following orders to wear masks. We also find evidence of distancing fatigue, but risk compensation results persist after accounting for distancing fatigue.

### Time at home

By the time face mask orders were issued, Americans were already experiencing distancing fatigue measured as the log of days since stay-at-home orders were issued, and time at home was already declining (Table 1). We find evidence of distancing fatigue in all states, but if anything, distancing fatigue is greatest in states that ultimately received face mask orders. Understanding distancing fatigue provides a baseline for behavioral shifts associated with mask mandates.

**Table 1:** Impacts of days since stay-at-home order issued on dwelling time at home (in min) during the mask mandate study periods for (a) with with-policy counties, and (b) all counties, with no-policy counties using the average date of mandates in state for study period construction. Standard errors are shown in parentheses, one, two, and three asterisk refer to 95%, 99%, 99.9% confidence level.

	± 14 days of face mask mandate (public)			± 14 days of face mask mandate (business)		
	Basic	Re-opening business	All re-opening	Basic	Re-opening business	All re-opening
**(a)**						
Dep Var: home dwell time						
log(days since stay-at-home policy)	− 36.82*	− 39.11*	− 37.16*	− 29.08*	− 26.91	− 26.71*
	(16.40)	(16.49)	(16.69)	(12.18)	(13.91)	(11.97)
End of stay-at-home policy	− 5.900	3.072	14.48*	3.609	0.0280	3.423
	(8.814)	(11.08)	(5.752)	(8.089)	(9.836)	(8.412)
log(new national cases)	50.34***	45.07***	45.35***	88.77***	91.99***	90.23***
	(9.131)	(10.86)	(9.976)	(16.22)	(18.71)	(17.54)
R square	0.884	0.884	0.885	0.873	0.873	0.874
Dep Var: log(home dwell time)						
log(days since stay-at-home policy)	− 0.0534*	− 0.0574*	− 0.0541*	− 0.0282	− 0.0238	− 0.0227
	(0.0228)	(0.0229)	(0.0227)	(0.0179)	(0.0223)	(0.0193)
End of stay-at-home policy	− 0.00989	0.00557	0.0250**	0.00151	− 0.00571	0.000231
	(0.0133)	(0.0175)	(0.00927)	(0.0130)	(0.0167)	(0.0145)
log(new national cases)	0.0749***	0.0658***	0.0672***	0.127***	0.134***	0.133***
	(0.0129)	(0.0149)	(0.0141)	(0.0233)	(0.0281)	(0.0262)
R square	0.865	0.865	0.867	0.854	0.854	0.856
N	64,739			73,405		
**(b)**						
Dep Var: home dwell time						
log(days since stay-at-home policy)	− 39.10*	− 41.38*	− 39.19*	− 23.92*	− 21.91	− 21.96
	(18.13)	(18.25)	(18.51)	(11.08)	(12.64)	(11.25)
End of stay-at-home policy	− 6.910	3.048	14.11*	4.320	0.917	5.316
	(8.603)	(10.76)	(5.614)	(7.163)	(9.054)	(8.308)
log(new national cases)	49.26***	44.26***	45.04***	89.29***	91.89***	90.56***
	(8.045)	(9.405)	(8.650)	(15.62)	(17.24)	(16.39)
R square	0.870	0.871	0.871	0.868	0.868	0.869
Dep Var: log(home dwell time)						
log(days since stay-at-home policy)	− 0.0616*	− 0.0654*	− 0.0617*	− 0.0214	− 0.0166	− 0.0164
	(0.0269)	(0.0272)	(0.0271)	(0.0172)	(0.0210)	(0.0190)
End of stay-at-home policy	− 0.0103	0.00645	0.0256**	0.00426	− 0.00372	0.00331
	(0.0131)	(0.0171)	(0.00894)	(0.0114)	(0.0151)	(0.0146)
log(new national cases)	0.0744***	0.0660***	0.0682***	0.132***	0.138***	0.138***
	(0.0116)	(0.0131)	(0.0126)	(0.0228)	(0.0261)	(0.0252)
R square	0.847	0.848	0.849	0.849	0.849	0.850
N	88,815			89,139		

**Figure 2:**
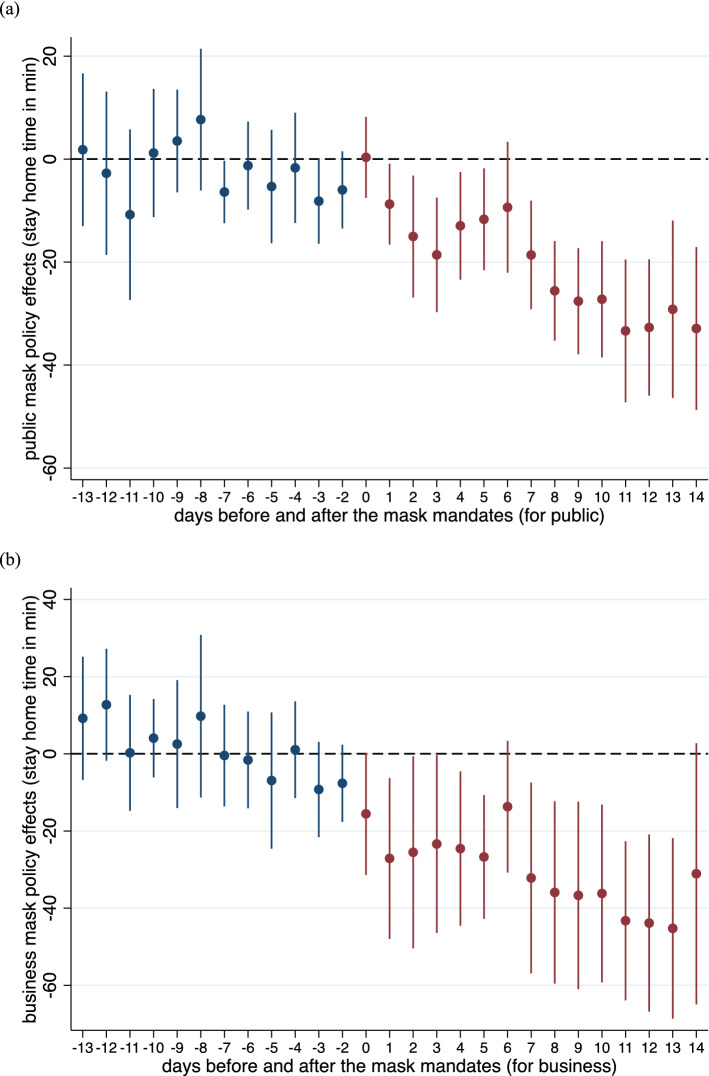
Dynamic mask mandate effects in 14 days before and after the mandates (**a**) for public and (**b**) for business. Blue is pre-mandate implementation and red is post-mandate implementation.

Americans appear to have reduced time at home following state mandates to wear face masks (Fig. 2). Estimates are stable across all specifications and range from a reduction in time at home from 10 to 12 min for mandates in public or 2–3.6%, and 16–24 min for mandates in business or 2–4% (Table 2). The estimate of distancing fatigue (day since stay-at-home orders) is robust to the inclusion of mask orders providing evidence that the face mask order effect is not confounded with distancing fatigue. Including no-policy counties with a study period constructed based on state-level average or earliest dates of mandates provides robust estimates (Table S1 and S2).

**Table 2:** Effects of face mask mandates for public and for business on dwelling time at home (in min) in 14 days before and after the mandates. (Only the with-policy counties).

	± 14 days of face mask mandate (public)			± 14 days of face mask mandate (business)		
	Basic	Re-opening business	All re-opening	Basic	Re-opening business	All re-opening
Dep Var: home dwell time						
Face mask mandate (public)	− 11.49**	− 11.09**	− 10.43**	**− **3.742	**− **4.138	**− **1.513
	**(3.458)**	**(3.463)**	**(3.553)**	(6.844)	(6.835)	(7.358)
Face mask mandate (business)	**− **11.01	**− **9.515	**− **8.558	− 16.92*	− 19.59*	− 24.14**
	(5.560)	(5.834)	(6.095)	**(6.544)**	**(7.283)**	**(7.317)**
log(days since stay-at-home policy)	**− **32.35*	**− **33.67*	**− **32.49*	**− **29.33*	**− **24.17	**− **23.95*
	(14.24)	(14.41)	(14.36)	(12.45)	(14.17)	(11.52)
End of stay-at-home policy	**− **0.914	1.412	12.59*	6.427	**− **1.641	**− **0.473
	(9.509)	(10.67)	(5.456)	(7.640)	(9.254)	(8.457)
log(new national cases)	47.91***	46.72***	47.42***	81.11***	87.49***	87.97***
	(8.663)	(9.436)	(9.312)	(11.66)	(13.04)	(12.64)
R square	0.885	0.885	0.886	0.874	0.875	0.876
Dep Var: log(home dwell time)						
Face mask mandate (public)	− 0.0181***	− 0.0171***	− 0.0163**	**− **0.00676	**− **0.00744	**− **0.00319
	**(0.00456)**	**(0.00459)**	**(0.00475)**	(0.0104)	(0.0104)	(0.0108)
Face mask mandate (business)	**− **0.0157	**− **0.0121	**− **0.0113	− 0.0244*	− 0.0290*	− 0.0357**
	(0.00860)	(0.00902)	(0.00948)	**(0.00950)**	**(0.0109)**	**(0.0108)**
log(days since stay-at-home policy)	**− **0.0471*	**− **0.0502*	**− **0.0479*	**− **0.0287	**− **0.0199	**− **0.0187
	(0.0197)	(0.0209)	(0.0203)	(0.0182)	(0.0226)	(0.0183)
End of stay-at-home policy	**− **0.00240	0.00326	0.0223*	0.00554	**− **0.00835	**− **0.00563
	(0.0145)	(0.0170)	(0.00892)	(0.0124)	(0.0156)	(0.0146)
log(new national cases)	0.0716***	0.0687***	0.0705***	0.117***	0.128***	0.130***
	(0.0117)	(0.0127)	(0.0127)	(0.0165)	(0.0190)	(0.0182)
R square	0.866	0.866	0.867	0.856	0.856	0.857
N	64,739			73,405		

Standard errors are presented in parentheses, one, two, and three asterisks refer to the 95%, 99% and 99.9% confidence level. Bold values are parameter estimates of primary interest, γ_1_ and γ_2_.

**Figure 3:**
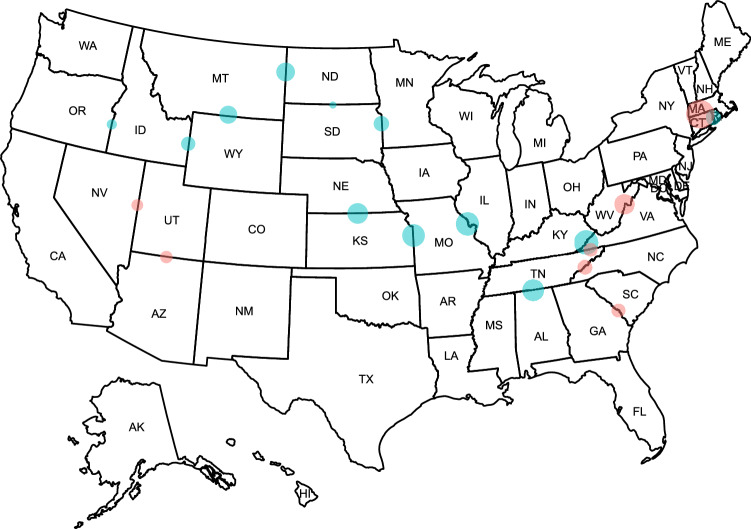
The heterogeneity of face mask mandate effects for use in businesses in terms of home dwell time (in minutes) across state borders. We use the differences-in-differences methods for each pair. The control states are no-policy states or have policies after 2 weeks of the corresponding treatment states. Blue dots imply the treated states demonstrate risk compensation behaviors and red dots imply evidence for the opposite effect. The dot sizes are the magnitudes of the coefficients. We only show the results for precisely estimated pairs using a 95% confidence level. The detailed table results are in Table S3.

We use the same constructed study periods for no-policy or late-policy states to examine face mask mandates for business for pairs of bordering states (Fig. 3 and Table S3). In the majority of states, we observe a reduction in time at home following business face mask mandates.

The effect of face masks is robust to including business reopening policies in the model. This is likely because few states closed or opened businesses within the window of analysis.

We investigate possible spillover effects of mask orders across the state border. We find that Americans in counties without mandates do not respond significantly to the bordering counties’ mask mandate (Table 3).

**Table 3:** Possible face mask mandates spillover effects to no-policy counties.

	± 14 days of in-effect counties’ face mask mandate (public)						± 14 days of in-effect counties’ face mask mandate (business)	
	Earliest neighboring counties		AVERAGE date in state		Earliest county in state		Earliest neighboring counties	
	Re-opening business	All re-opening	Re-opening business	All re-opening	Re-opening business	All re-opening	Re-opening business	All re-opening
Dep Var: home dwell time								
Face mask mandate (public)	**−** 7.827*	**−** 7.106*	**4.781**	**4.821**	**−** **2.634**	**2.685**	− 7.042	− 5.753
	**(3.022)**	**(3.114)**	**(6.882)**	**(6.887)**	**(9.338)**	**(8.090)**	(4.823)	(3.811)
Face mask mandate (business)	− 0.618	4.854	0	0	0	0	**1.396**	**3.411**
	(3.597)	(3.042)	(.)	(.)	(.)	(.)	**(8.880)**	**(7.690)**
log(days since stay-at-home policy)	− 0.527	0.504	− 18.58	0.316	1.572	3.368	25.09	51.54
	(9.256)	(8.994)	(48.33)	(41.59)	(8.216)	(5.668)	(22.72)	(29.09)
End of stay-at-home policy	− 28.24	− 8.340	0	0	0	0	− 29.82*	− 34.86**
	(18.24)	(17.04)	(.)	(.)	(.)	(.)	(11.70)	(9.349)
log(new national cases)	57.04***	50.79***	15.04	8.628	22.66	9.506	85.53**	82.60**
	(8.092)	(8.222)	(17.36)	(17.90)	(10.59)	(10.62)	(14.73)	(16.12)
R square	0.842	0.844	0.813	0.813	0.826	0.828	0.834	0.836
Dep Var: log(home dwell time)								
Face mask mandate (public)	**−** 0.0137*	**−** 0.0125*	**0.00752**	**0.00746**	**0.000274**	**0.00883**	− 0.00802	− 0.00557
	**(0.00474)**	**(0.00503)**	**(0.0111)**	**(0.0112)**	**(0.0159)**	**(0.0143)**	(0.00674)	(0.00578)
Face mask mandate (business)	0.00143	0.0101	0	0	0	0	**0.00177**	**0.00526**
	(0.00621)	(0.00519)	(.)	(.)	(.)	(.)	**(0.0130)**	**(0.0118)**
log(days since stay-at-home policy)	− 0.00157	0.0000538	− 0.0555	− 0.0219	0.000764	0.00322	0.0453	0.0904
	(0.0170)	(0.0174)	(0.0896)	(0.0785)	(0.0128)	(0.00873)	(0.0340)	(0.0427)
End of stay-at-home policy	− 0.0411	− 0.0119	0	0	0	0	− 0.0367	− 0.0477**
	(0.0305)	(0.0312)	(.)	(.)	(.)	(.)	(0.0163)	(0.0120)
log(new national cases)	0.0813***	0.0716***	0.0125	− 0.000443	0.0355	0.0135	0.123**	0.117**
	(0.0136)	(0.0139)	(0.0289)	(0.0299)	(0.0190)	(0.0187)	(0.0220)	(0.0236)
R square	0.824	0.826	0.793	0.793	0.804	0.806	0.830	0.832
N	12,478		21,611		22,201		5618	

Standard errors are presented in parentheses, one, two, and three asterisks refer to the 95%, 99% and 99.9% confidence level. Bold values are the parameter estimates of primary interest γ_1_ and γ_2_.

### Points of interest visitation

**Figure 4:**
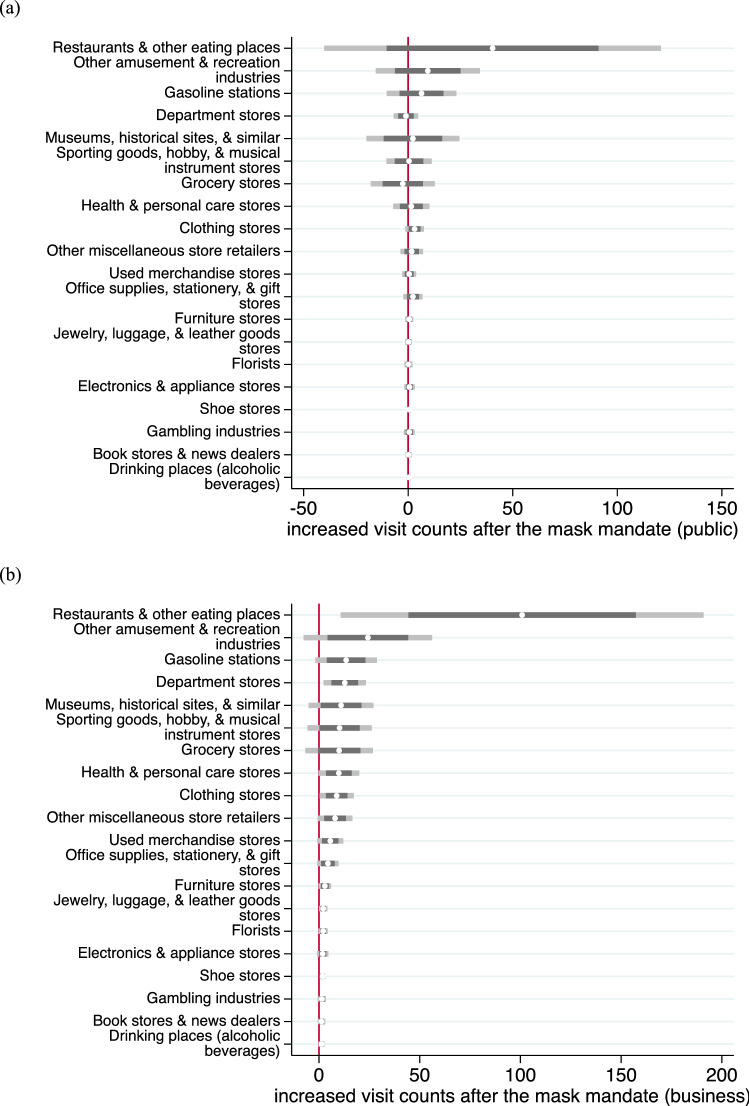
Estimates of increase in visits to a sample of specific types of locations following the face mask mandates (**a**) for public and (**b**) for business with 95% (dark gray) and 99.75% (grey) Bonferroni confidence intervals. A Bonferroni correction suggests that the 99.75% confidence interval is what should be used conservatively for a 5% probability of a type I error, to address concerns about multiple testing. Parks are included in Museums, historic sites, and similar institutions. Golf courses are included in Other amusement and recreation industries.

Americans increased trips to a variety of places in the weeks following the mask mandate (Fig. 4). The greatest effects were for restaurants and other eating places, which could be dominated by take-out pick-ups because we look at the count of visits and not time on site. Fisher (2020) reports that adults testing positive for COVID-19 were twice as likely to have dined at a restaurant relative to adults with negative tests. The next most affected locations were recreation locations, which includes parks, health and personal care, and florist, which may include gardening stores. Grocery stores and various merchant wholesalers did not appear to receive increased trips following the mask orders.

Overall, the increase in out-of-the-house activity appears to expose people to a mix of sites with different risk characteristics. Variation in site risk characteristics implies that it is difficult to know if the net effect of mask orders and risk compensation increased or decreased transmission risk relative to staying home. The mechanism by which mask orders could reduce transmission is that they could reduce contacts among maskless individuals similar to the way that adding immune individuals can reduce transmission in an otherwise identical population^11^. Furthermore, the net effect may vary by geography.

### Addressing potential concerns

One possibility is that governors and local leaders simply run to the front of the crowd. The assumption would be that leaders know people will start going out. Therefore, they implement face mask orders to signal it is ok to start going out. If this were the case, it is unlikely that there would be a systematic break, and a substantial number of governors relaxed stay-at-home orders directly. Furthermore, such a concern simultaneously gives the governors a large amount of credit for political astuteness and treats their actions with a high degree of cynicism. Both assumptions seem unmerited in the fog of the COVID-19 crisis.

We analyze the effect of the mask orders defined by the implementation date and not the announcement. It is possible that policy announcements or earlier CDC and WHO announcements signaled to the public that it is safe to resume public interaction with a mask. Such anticipatory behavior would attenuate our estimates because the pre-policy period would be contaminated by the behavioral response that we associate with the face mask orders. Still, we find a robust decrease in time spent at home and a robust increase in trips to public places following the implementation of face mask requirements. Furthermore, we find no effect in states without mask orders to the orders of neighboring states.

## Discussion

Americans increased visitation to public locations and reduced their time spent at home even as COVID-19 cases rose in much of the United States. Some of this was distancing fatigue. However, our results suggest that mask orders provide a sense of protection. When everyone was expected to wear a mask, people perceived a lower risk associated with leaving home and visiting public locations. This led people to substitute away from other non-pharmaceutical interventions like avoiding time in public. The net effect of these behaviors on public health outcomes depends on the relative effectiveness of masks and other behaviors in reducing transmission. Recent evidence suggests that the net effect of masks has been to reduce transmission of SARS-CoV-2^24^, but other explanations remain possible.

Evidence suggests that staying home effectively reduced transmission. One concern with that conclusion is that it is easier to observe staying home behavior than handwashing, physical spacing, and face mask-wearing. For face masks to be effective, they must be used consistently (Figure S4) and correctly. This includes having a tight seal, which requires things like a clean shave, having one’s nose in the mask, and leaving the mask on while talking to someone. One need only look at images of mask-wearing in public to conclude that a non-trivial share of mask wearers are not wearing them correctly or that the masks themselves are of questionable quality (i.e., bandannas). It is certainly possible that misused masks do not increase transmission, and may reduce it, holding other behaviors constant. To assess the effectiveness of face mask mandates, we need to understand the impact of face mask mandates and face mask use on other behaviors.

Our results suggest that wearing a mask, given real compliance levels, needs to be as effective as individuals, reducing their average time out of the house by approximately 1.6–3.% or 11–24 min. Bayham et al.^25^ found that voluntary behavioral change of a similar magnitude reduced 2009 H1N1 swine flu cases on the order of 10%. The swine flu estimate is likely a lower bound for the value of additional time at home for reducing COVID-19 cases, as COVID-19 appears more transmissible. Are face masks in the general public equivalent to the average individual staying home an additional 11–24 min a day—likely one less trip somewhere? This is a challenging empirical question that still needs answering. However, systematic data on individual behaviors similar to the smart device data do not exist for mask wearing and other protective measures, making it difficult to rule out other explanations of declining cases in some areas. Cases increased in some states and decreased in other states despite mask orders. Nevertheless, our results provide a benchmark for future testing of the effectiveness of face masks in the general public.

Our results should not be used as a justification for discouraging face mask use. Rather, extreme care must be taken when suggesting new behaviors that may be helpful, in order to avoid replacing behaviors that are known to be helpful. The message to wear a face mask in public is at least suggestive that it is safe to resume public interactions with a mask. However, time in public is still riskier than time at home and can still enable transmission from asymptomatic individuals.

An alternative interpretation of our results is that mask orders can help inspire confidence to encourage people to return to commerce if people overestimate the local risk of infection. If policy makers believe that COVID-19 is under control, and want to encourage a return to commerce, then mask orders might help stimulate the local economy. This would be especially true if people continue to feel unsafe in public spaces because they overestimate infection risk.

Overall, the results suggest some risk compensation, with trips directed to one of the highest risk locations. However, our state comparisons do raise identification questions beyond the net effect. Evaluating COVID-19 policies is challenging because many policies were initiated in short-order and policies likely interact^19^. This feature of evaluating non-pharmaceutical interventions means that each study can add a weight of evidence, but that no study will provide a definitive answer to the relative effectiveness of any one policy intervention.

There can be risk compensation and the net effect can be beneficial^26^. Requiring seat belts likely outweighs the damage from riskier driving and energy efficiency reduces carbon emissions even if it leads to more device use^27^. Even encouraging condom use has likely prevented more cases of HIV, then the risk compensation generated. At present it is unclear whether the risk compensation associated with masks results in a net benefit or net cost. However, the difference between a few trips with a mask and staying home, spread across the entire population could be the difference between the reproductive rate of the pathogen (R(t)) exceeding one, and renewed exponential growth, and a reproductive rate less than one and containing the epidemic. If people must go out, then it is advisable to wear a mask. The fact that people now own masks is likely making them more likely to even consider a trip out. On one hand, these marginal trips could make the epidemic more difficult to bring under control, on the other they may alleviate the economic stress creating space to control the pandemic. Either way, it is important to understand the substitution effects among non-pharmaceutical responses.

## Supplementary Information

Supplementary Information

## Data Availability

Code available at https://github.com/youpeiyan/face_mask_mandate with instructions for acquiring data.
