# Supplementary material for: Risk compensation and face mask mandates during the COVID-19 pandemic
Source: Sci Rep. 2021 Feb 4;11:3174. doi: 10.1038/s41598-021-82574-w (PMC7862259; doi:10.1038/s41598-021-82574-w)
Supplement: Supplementary file 1 — Supplementary Information [file 41598_2021_82574_MOESM1_ESM.pdf]

Title: Supplemental Information for Risk Compensation and Face Mask Mandates During the COVID-19 Pandemic

Authors: Youpei Yan[1], Jude Bayham[2], Aaron Richter[1], Eli P. Fenichel[1]

Affiliations:
[1]Yale University, 195 Prospect St. New Haven, CT, 06511, youpei.yan@yale.edu. eli.fenichel@yale.edu, aaron.richter@yale.edu

[2]Colorado State University, B303 Clark Bldg, Fort Collins, CO jude.bayham@colostate.edu

Corresponding Author: Eli Fenichel

Abstract:
Face masks are an important component in controlling COVID-19, and policy orders to wear masks are common. However, behavioral responses are seldom additive, and exchanging one protective behavior for another could undermine the COVID-19 policy response. We use SafeGraph smart device location data and variation in the date that US states and counties issued face mask mandates as a set of natural experiments to investigate risk compensation behavior. We compare time at home and the number of visits to public locations before and after face mask orders conditional on multiple statistical controls. We find that face mask orders lead to risk compensation behavior. Americans subject to the mask orders spend 11-24 fewer minutes at home on average and increase visits to some commercial locations – most notably restaurants, which are a high-risk location. It is unclear if this would lead to a net increase or decrease in transmission. However, it is clear that mask orders would be an important part of an economic recovery if people otherwise overestimate the risk of visiting public places.

Keywords: social distancing; non-pharmaceutical intervention; epidemic; pandemic

SUPPLEMENTAL INFORMATION

(a)

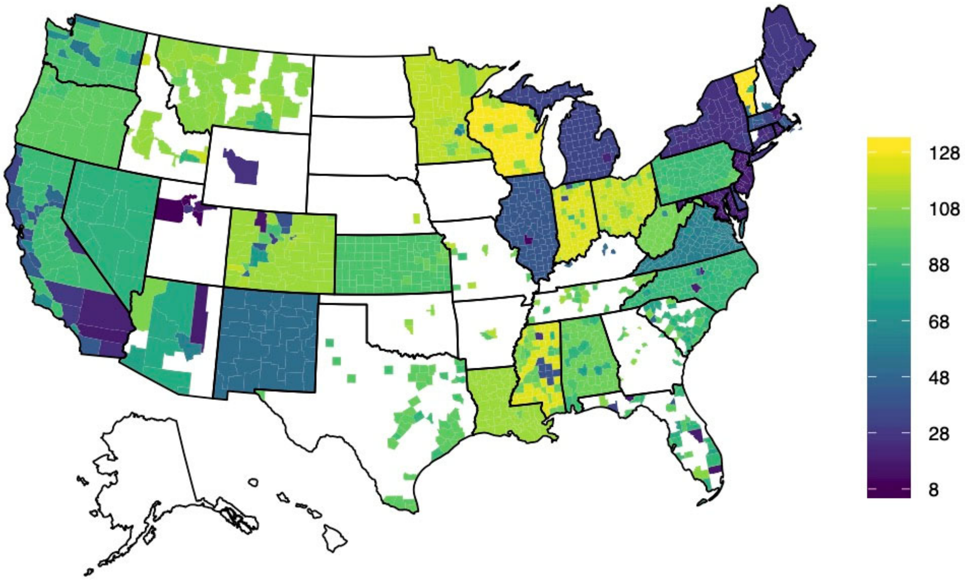

(b)

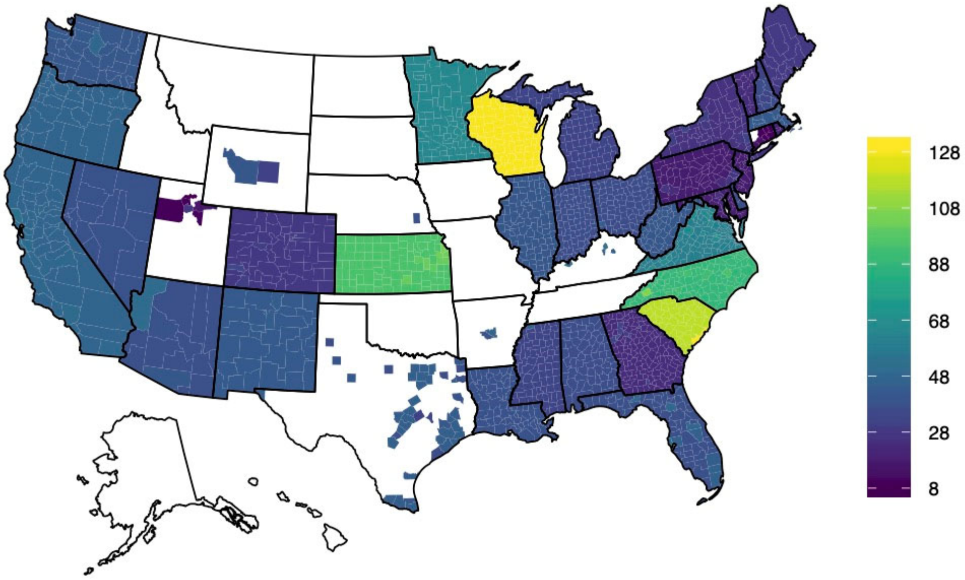

Figure S1. Number of days that face mask mandates are in effect for (a) public and (b) business since the start date of the first stay-at-home policy. Maps generated using R 3.6.2 all code at https://github.com/youpeiyan/face_mask_mandate.

(a)

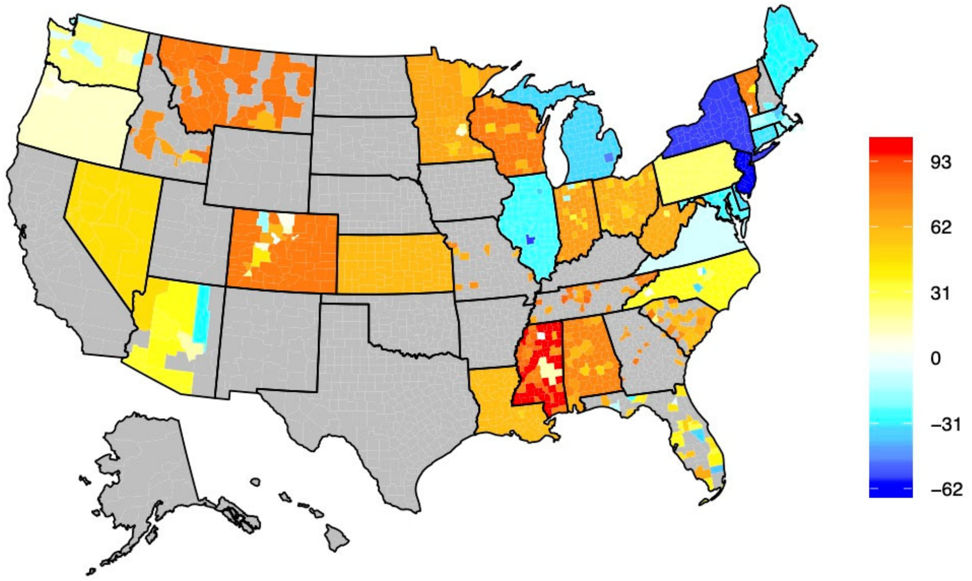

(b)

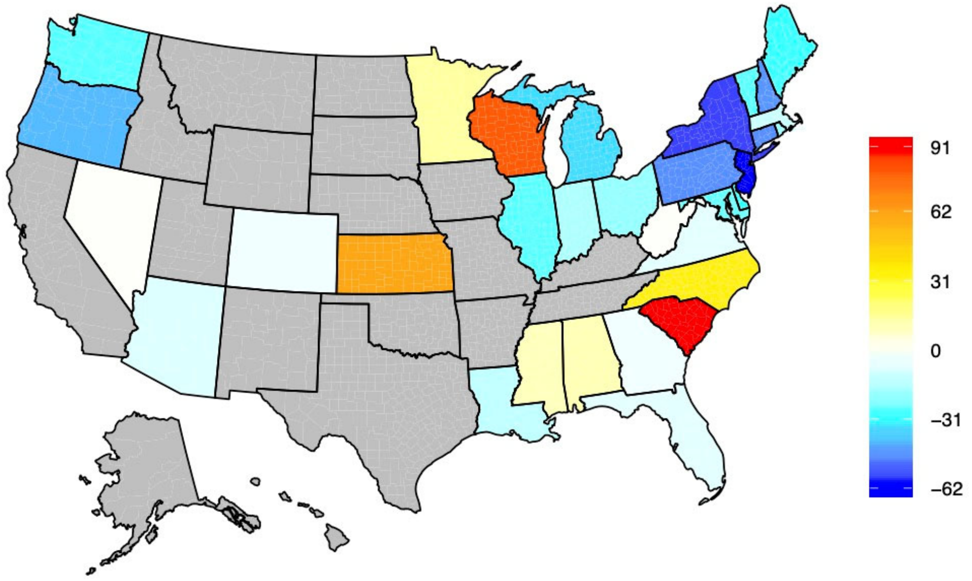

Figure S2. Number of days that face mask mandates are in effect for (a) public and (b) business since the end date of the first stay-at-home policy. Negative values mean that face mask mandates issued before the end of the stay-at-home policy. Maps generated using R 3.6.2 all code at https://github.com/youpeiyan/face_mask_mandate.

(a)

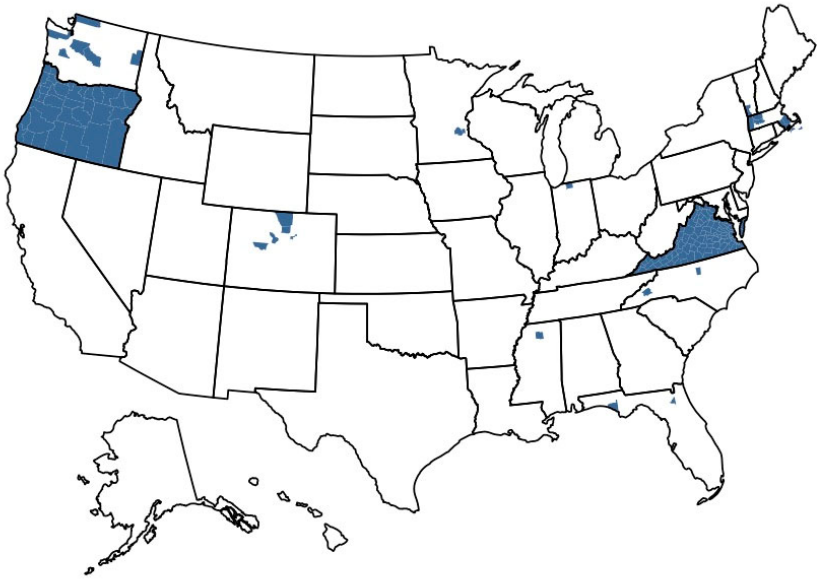

(b)

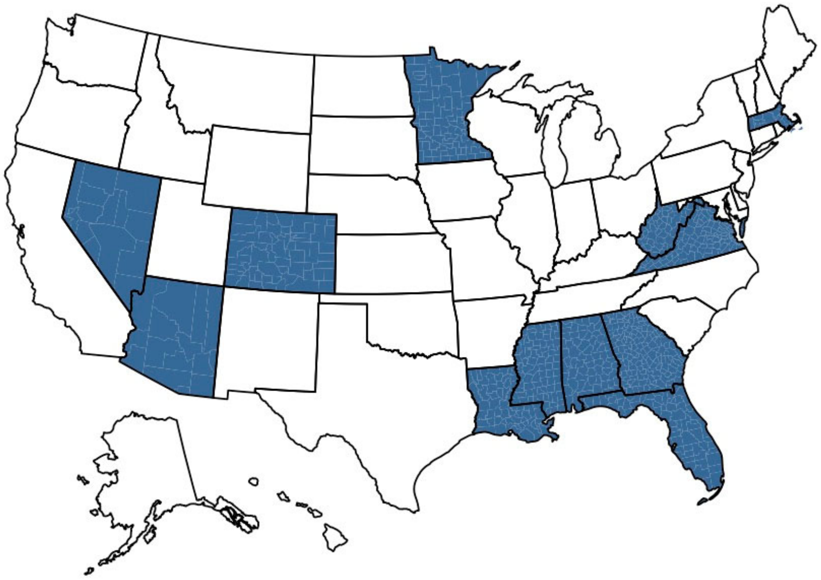

Figure S3. Counties that issued face mask mandates in a +/- 14 day window of the end of stay-at-home policies (a) for public and (b) for business. Maps generated using R 3.6.2 all code at https://github.com/youpeiyan/face_mask_mandate.

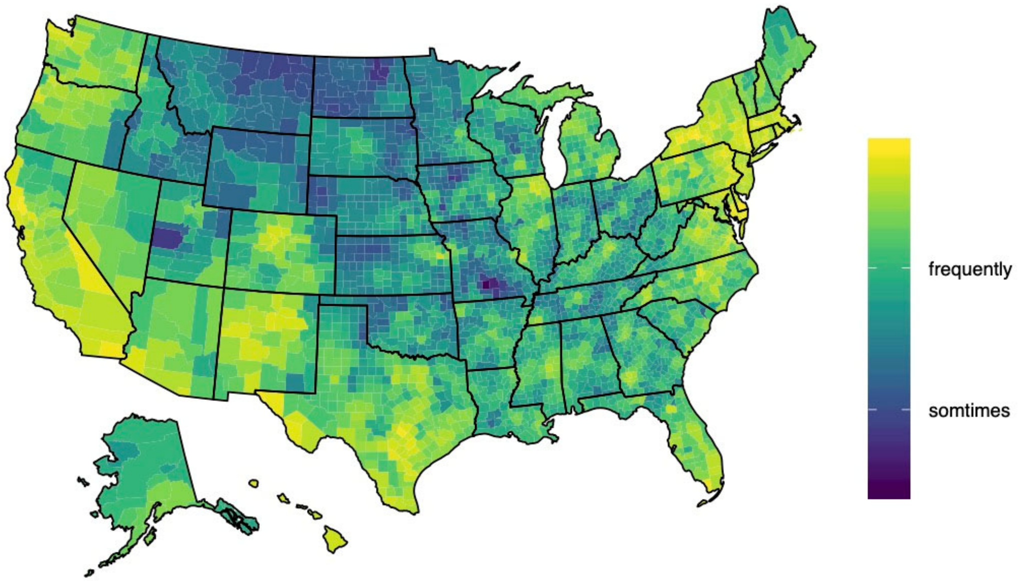

Figure S4. Mask wearing frequency from “rarely” (dark purple) to “always” (yellow) based on estimates from The New York Times, based on roughly 250,000 interviews conducted by Dynata from July 2 to July 14. (https://github.com/nytimes/covid-19-data/tree/master/mask-use). Maps generated using R 3.6.2 all code at https://github.com/youpeiyan/face_mask_mandate.

Table S1. Effects of face mask mandates for public and for business on dwelling time at home (in min) in the 14 days before and after the mandates for with-policy counties and no-policy counties. For no-policy counties, the study periods are chosen based on the **earliest** county-level policy in state; if there is no policy in state, then the **earliest** policy in the country.

	+/-14 days of face mask mandate (public)			+/-14 days of face mask mandate (business)		
Dep Var: dwell time	basic	re-opening business	all re-opening	basic	re-opening business	all re-opening
face mask mandate	**-11.54*** **	**-11.31*** **	**-10.65*** **	-7.088	-7.393	-4.458
(public)	**(2.970)**	**(2.996)**	**(2.918)**	(7.548)	(7.103)	(8.161)
face mask mandate	-15.86*	-13.85	-13.31	**-28.31*** **	**-31.36*** **	**-36.28*** **
(business)	(6.765)	(8.048)	(8.511)	**(5.146)**	**(5.889)**	**(6.820)**
log(days since	-8.005	-8.900	-8.030	-8.571*	-7.451	-7.280*
stay-at-home policy)	(15.57)	(16.15)	(15.21)	(4.125)	(4.003)	(3.542)
end of stay-at-home	-2.211	1.094	10.08*	0.509	-7.856	-6.997
policy	(9.148)	(9.737)	(4.380)	(6.658)	(8.109)	(7.994)
log(new national cases)	39.67***	38.97***	37.89***	95.71***	101.2***	100.1***
	(8.133)	(8.320)	(8.326)	(8.919)	(9.354)	(9.850)
R square	0.869	0.869	0.870	0.864	0.864	0.865

	+/-14 days of face mask mandate (public)			+/-14 days of face mask mandate (business)		
Dep Var: log(dwell time)	basic	re-opening business	all re-opening	basic	re-opening business	all re-opening
face mask mandate	**-0.0195*** **	**-0.0190*** **	**-0.0180*** **	-0.0127	-0.0132	-0.00866
(public)	**(0.00458)**	**(0.00460)**	**(0.00450)**	(0.0109)	(0.0103)	(0.0115)
face mask mandate	-0.0223*	-0.0184	-0.0179	**-0.0393*** **	**-0.0442*** **	**-0.0517*** **
(business)	(0.00987)	(0.0112)	(0.0119)	**(0.00753)**	**(0.00893)**	**(0.0101)**
log(days since	-0.0183	-0.0200	-0.0188	-0.00901	-0.00720	-0.00694
stay-at-home policy)	(0.0240)	(0.0251)	(0.0239)	(0.00752)	(0.00740)	(0.00690)
end of stay-at-home	-0.00299	0.00344	0.0197**	-0.000704	-0.0142	-0.0117
policy	(0.0138)	(0.0156)	(0.00730)	(0.0113)	(0.0139)	(0.0143)
log(new national cases)	0.0616***	0.0603***	0.0589***	0.144***	0.153***	0.152***
	(0.0125)	(0.0126)	(0.0127)	(0.0129)	(0.0138)	(0.0140)
R square	0.848	0.848	0.849	0.845	0.845	0.847
N		88615			89172	

Table S2. Effects of face mask mandates for public and for business on dwelling time at home (in min) in 14 days before and after the mandates for with-policy counties and no-policy counties. For no-policy counties, the study periods are chosen based on the **average** date of county-level policy in state; if there is no policy in state, then the **average** date of policy in the country.

	+/-14 days of face mask mandate (public)			+/-14 days of face mask mandate (business)		
Dep Var: dwell time	basic	re-opening business	all re-opening	basic	re-opening business	all re-opening
face mask mandate	**-8.879****	**-8.482****	**-7.761***	-4.477	-4.797	-5.788
(public)	**(3.084)**	**(3.028)**	**(3.059)**	(6.696)	(6.678)	(7.185)
face mask mandate	-11.96*	-9.759	-9.288	**-14.36***	**-16.31***	**-18.40****
(business)	(5.641)	(6.055)	(6.347)	**(5.619)**	**(6.350)**	**(6.075)**
log(days since	-32.09*	-34.09*	-32.54*	-23.44*	-19.03	-18.71
stay-at-home policy)	(15.31)	(15.45)	(15.37)	(11.29)	(12.63)	(10.75)
end of stay-at-home	-1.435	1.974	12.77*	6.315	-0.718	2.200
policy	(9.361)	(10.22)	(5.251)	(6.839)	(8.912)	(8.566)
log(new national cases)	44.75***	43.42***	44.50***	86.26***	91.34***	92.73***
	(7.703)	(8.223)	(8.085)	(11.96)	(12.96)	(12.38)
R square	0.871	0.871	0.872	0.869	0.869	0.870

	+/-14 days of face mask mandate (public)			+/-14 days of face mask mandate (business)		
Dep Var: log(dwell time)	basic	re-opening business	all re-opening	basic	re-opening business	all re-opening
face mask mandate	**-0.0153*****	**-0.0145****	**-0.0135****	-0.00826	-0.00886	-0.0104
(public)	**(0.00436)**	**(0.00430)**	**(0.00436)**	(0.0103)	(0.0104)	(0.0106)
face mask mandate	-0.0163	-0.0118	-0.0119	**-0.0191***	**-0.0228***	**-0.0267****
(business)	(0.00868)	(0.00920)	(0.00962)	**(0.00838)**	**(0.00962)**	**(0.00906)**
log(days since	-0.0514*	-0.0555*	-0.0525*	-0.0209	-0.0127	-0.0118
stay-at-home policy)	(0.0230)	(0.0242)	(0.0237)	(0.0176)	(0.0212)	(0.0182)
end of stay-at-home	-0.00195	0.00500	0.0237**	0.00688	-0.00624	-0.00142
policy	(0.0143)	(0.0163)	(0.00845)	(0.0109)	(0.0148)	(0.0150)
log(new national cases)	0.0680***	0.0653***	0.0677***	0.129***	0.138***	0.142***
	(0.0106)	(0.0112)	(0.0113)	(0.0177)	(0.0195)	(0.0187)
R square	0.848	0.849	0.849	0.850	0.851	0.852
N		88815			89139	

Table S3. The heterogeneity of face mask mandate (for business) effects for dwell home time (in minutes) between bordering states. (Figure 3's results. The left is the ordered-state or first ordered-state, N is for total observations.) The p-value refers to the probability of falsely rejecting the evidence of parallel when it should not be rejected. A low p-value suggests the parallel pre-trends assumption is not supported. We suggest that pairs below the dashed line do not have sufficiently parallel pre-trends for the difference-in-difference design. Precise estimates supporting risk compensation are in blue. Precise estimates supporting complementaries between masks and stay home are in red.

	dwell-time		log(dwell-time)			p-val:
Pair of states	mandate for business	(sd)	mandate for business	(sd)	N	parallel trend test
WI_IA	-6.886	(3.737)	-0.0219**	(0.00723)	5376	0.98
KS_MO	-60.48***	(3.297)	-0.0749***	(0.00518)	3538	0.96
DC_VA	-11.13	(8.635)	-0.0189	(0.0125)	2204	0.94
WA_ID	2.201	(4.259)	-0.000308	(0.00719)	5509	0.93
VA_TN	15.61***	(3.097)	0.0223***	(0.00467)	7511	0.93
UT_NV	11.95***	(3.337)	0.0189***	(0.00510)	4408	0.91
NC_TN	18.95**	(6.873)	0.0350**	(0.0127)	4457	0.84
NM_OK	-41.54	(29.11)	-0.0678	(0.0478)	1256	0.83
WY_ID	-18.59*	(9.154)	-0.0297	(0.0156)	2255	0.80
CT_RI	-19.88***	(3.066)	-0.0245***	(0.00463)	4698	0.80
RI_MA	-3.301	(2.677)	-0.00527	(0.00390)	7366	0.80
WY_SD	20.76	(10.92)	0.0276	(0.0149)	5829	0.79
ND_SD	-7.066*	(3.542)	-0.00783	(0.00540)	4930	0.79
WV_VA	45.45***	(5.418)	0.0669***	(0.00911)	5973	0.75
VT_MA	10.30	(7.552)	0.0212	(0.0145)	3984	0.62
NY_MA	-35.92	(31.20)	-0.0406	(0.0499)	3770	0.61
AL_TN	-53.22***	(8.420)	-0.0835***	(0.0117)	377	0.60
CT_MA	102.1***	(18.61)	0.192***	(0.0377)	2187	0.58
MD_VA	-7.016	(10.13)	0.00143	(0.0204)	2987	0.58
NE_KS	-47.50***	(10.94)	-0.0544**	(0.0174)	2291	0.52
GA_SC	16.60***	(2.771)	0.0262***	(0.00427)	5131	0.50
OR_ID	-9.169**	(3.111)	-0.0138*	(0.00570)	9446	0.49
NJ_DE	22.15	(11.50)	0.0371	(0.0213)	6637	0.49
WY_MT	-32.97***	(6.589)	-0.0699***	(0.00996)	638	0.49
KY_VA	-69.52***	(17.90)	-0.122***	(0.0343)	1706	0.42
ND_MT	-36.67***	(9.365)	-0.0434**	(0.0149)	4437	0.40
UT_ID	11.38	(10.14)	0.0151	(0.0183)	5274	0.35
MN_WI	-30.78	(24.36)	-0.0229	(0.0371)	6815	0.34
CO_KS	-18.96	(27.44)	-0.0364	(0.0471)	1506	0.34
MN_IA	-5.054	(5.591)	-0.00184	(0.0106)	4779	0.22
MN_SD	-22.41***	(4.981)	-0.0480***	(0.0111)	4270	0.22
NE_MO	5.114	(4.722)	0.0103	(0.00831)	5336	0.18
GA_TN	-10.45	(16.10)	-0.00881	(0.0211)	551	0.18
PA_WV	-1.467	(7.343)	-0.00475	(0.0131)	6376	0.15
MI_WI	18.42	(11.16)	0.0265	(0.0154)	6293	0.14
UT_AZ	13.45*	(5.613)	0.0163	(0.0101)	3130	0.11
NE_IA	5.870	(11.98)	0.0121	(0.0207)	2384	0.07

AR_TN	-16.20	(9.102)	-0.0274	(0.0178)	3288	0.07
IL_MO	-63.35***	(6.432)	-0.0842***	(0.0106)	4491	0.05
NE_SD	1.652	(3.688)	0.00649	(0.00630)	5654	0.04
IL_IA	47.71***	(8.590)	0.0818***	(0.0165)	2490	0.04
KY_TN	23.50***	(3.763)	0.0403***	(0.00675)	4233	0.02
TX_OK	-34.79***	(10.37)	-0.0634**	(0.0196)	2281	0.02
AR_MO	-13.72	(11.42)	-0.0209	(0.0174)	5045	0.02
ND_MN	-51.77***	(10.35)	-0.0887***	(0.0154)	812	0.01
IL_WI	-13.99***	(4.037)	-0.0189**	(0.00620)	5510	0.01
UT_WY	-43.91***	(6.852)	-0.0660***	(0.0112)	4887	0.00
KY_MO	-11.49***	(3.370)	-0.0227**	(0.00720)	4592	0.00
MS_TN	-1.934	(2.815)	-0.00676	(0.00414)	5945	0.00

Table S4. Estimates of increase in visits to a sample of specific types of locations following the face mask mandates (a) for public and (b) for business (Figure 4's 95% results).

	+/-14 days of facial mask mandate (business)			
	coef. (sd) of facial mask mandate (business)		Bonferroni coef. (sd) of facial mask mandate (business)	
Restaurants & other eating places	100.8***	(27.96)	100.8*	(27.96)
Other amusement & recreation industries	24.29*	(9.911)	24.29	(9.911)
Gasoline stations	13.49**	(4.753)	13.49	(4.753)
Department stores	12.81***	(3.266)	12.81**	(3.266)
Museums, historical sites, & similar institutions	10.96*	(5.000)	10.96	(5.000)
Sporting goods, hobby, & musical instrument stores	10.22*	(4.943)	10.22	(4.943)
Grocery stores	10.02	(5.213)	10.02	(5.213)
Health & personal care stores	9.878**	(3.150)	9.878	(3.150)
Clothing stores	8.783**	(2.645)	8.783*	(2.645)
Other miscellaneous store retailers	7.993**	(2.684)	7.993	(2.684)
Used merchandise stores	5.611**	(2.007)	5.611	(2.007)
Office supplies, stationery, & gift stores	4.443*	(1.647)	4.443	(1.647)
Furniture stores	2.929**	(0.970)	2.929	(0.970)
Jewelry, luggage, & leather goods stores	2.121**	(0.655)	2.121*	(0.655)
Florists	2.067**	(0.721)	2.067	(0.721)
Electronics & appliance stores	1.866*	(0.885)	1.866	(0.885)
Shoe stores	1.830***	(0.415)	1.830**	(0.415)
Gambling industries	1.419*	(0.642)	1.419	(0.642)
Book stores & news dealers	1.295*	(0.579)	1.295	(0.579)
Drinking places (alcoholic beverages)	1.250**	(0.447)	1.250	(0.447)
N	73385			

	+/-14 days of facial mask mandate (public)			
	coef. (sd) of facial mask mandate (public)		Bonferroni coef. (sd) of facial mask mandate (public)	
Restaurants & other eating places	40.39	(25.18)	40.39	(25.18)
Other amusement & recreation industries	9.388	(7.798)	9.388	(7.798)
Gasoline stations	6.399	(5.215)	6.399	(5.215)
Department stores	-1.058	(1.837)	-1.058	(1.837)
Museums, historical sites, & similar institutions	2.283	(6.966)	2.283	(6.966)
Sporting goods, hobby, & musical instrument stores	0.428	(3.380)	0.428	(3.380)
Grocery stores	-2.561	(4.785)	-2.561	(4.785)
Health & personal care stores	1.510	(2.720)	1.510	(2.720)
Clothing stores	3.078*	(1.404)	3.078	(1.404)
Other miscellaneous store retailers	1.716	(1.702)	1.716	(1.702)

Used merchandise stores	0.491	(1.048)	0.491	(1.048)
Office supplies, stationery, & gift stores	2.292	(1.438)	2.292	(1.438)
Furniture stores	0.506	(0.597)	0.506	(0.597)
Jewelry, luggage, & leather goods stores	0.200	(0.508)	0.200	(0.508)
Florists	0.175	(0.605)	0.175	(0.605)
Electronics & appliance stores	0.634	(0.790)	0.634	(0.790)
Shoe stores	0.608*	(0.301)	0.608	(0.301)
Gambling industries	0.518	(0.811)	0.518	(0.811)
Book stores & news dealers	0.192	(0.465)	0.192	(0.465)
Drinking places (alcoholic beverages)	0.167	(0.348)	0.167	(0.348)
N	65167			
